# Literary Notes

**Published:** 1910-01-22

**Authors:** 


### Literary Notes.

Mr. P. D. Malloch, of Perth, is publishing through Messrs. A. and C. Black a book on the "Life History and Habits of the Salmon, Sea-trout, Trout, and other Fresh-water Fish." From his connection with the Tay Salmon Fisheries Company the author has had unique opportunities of mastering the subject, and has been able to clear up many points that have remained a mystery until now, by the marking of smolts and their recapture as grilse and salmon. The study of scales also forms an interesting section of the book, which will be profusely illustrated.

There will be published immediately by Messrs. Black a translation, for the first time in English, of Spinoza's "Short Treatise on God, Man, and His Well-being." Professor A. Wolf has made the translation and has edited it with an introduction and commentary. The "Short Treatise" contains the essentials of Spinoza's philosophy in an easier and more readable form than the "Ethica," and is therefore more suitable to enable the beginner and the general reader to make a first-hand acquaintance with Spinoza. At the same time, while furnishing an excellent introduction to Spinoza, this volume will also provide more advanced students with a perfectly reliable version of the "Short Treatise" which is indispensable for the study of the inner development of Spinoza's philosophy.

